# Functional connectivity-based parcellation and connectome of cortical midline structures in the mouse: a perfusion autoradiography study

**DOI:** 10.3389/fninf.2014.00061

**Published:** 2014-06-11

**Authors:** Daniel P. Holschneider, Zhuo Wang, Raina D. Pang

**Affiliations:** ^1^Department of Psychiatry and Behavioral Sciences, University of Southern CaliforniaLos Angeles, CA, USA; ^2^Departments of Neurology, Cell and Neurobiology, Biomedical Engineering, University of Southern CaliforniaLos Angeles, CA, USA

**Keywords:** prelimbic cortex, infralimbic cortex, cingulate cortex, retrosplenial cortex, fear, functional connectome

## Abstract

Rodent cortical midline structures (CMS) are involved in emotional, cognitive and attentional processes. Tract tracing has revealed complex patterns of structural connectivity demonstrating connectivity-based integration and segregation for the prelimbic, cingulate area 1, retrosplenial dysgranular cortices dorsally, and infralimbic, cingulate area 2, and retrosplenial granular cortices ventrally. Understanding of CMS functional connectivity (FC) remains more limited. Here we present the first subregion-level FC analysis of the mouse CMS, and assess whether fear results in state-dependent FC changes analogous to what has been reported in humans. Brain mapping using [^14^C]-iodoantipyrine was performed in mice during auditory-cued fear conditioned recall and in controls. Regional cerebral blood flow (CBF) was analyzed in 3-D images reconstructed from brain autoradiographs. Regions-of-interest were selected along the CMS anterior-posterior and dorsal-ventral axes. In controls, pairwise correlation and graph theoretical analyses showed strong FC within each CMS structure, strong FC along the dorsal-ventral axis, with segregation of anterior from posterior structures. Seed correlation showed FC of anterior regions to limbic/paralimbic areas, and FC of posterior regions to sensory areas–findings consistent with functional segregation noted in humans. Fear recall increased FC between the cingulate and retrosplenial cortices, but decreased FC between dorsal and ventral structures. In agreement with reports in humans, fear recall broadened FC of anterior structures to the amygdala and to somatosensory areas, suggesting integration and processing of both limbic and sensory information. Organizational principles learned from animal models at the mesoscopic level (brain regions and pathways) will not only critically inform future work at the microscopic (single neurons and synapses) level, but also have translational value to advance our understanding of human brain architecture.

## Introduction

The importance of building brain connectomes to help understand brain structure and function has received increasing attention (Sporns et al., 2005). Multiple projects are underway to construct structural connectomes for the rodent (Bota et al., 2012) (see also the Mouse Connectome Project, http://www.mouseconnectome.org; and the mouse connectivity database in the Allen Brain Atlas, http://connectivity.brain-map.org) and human brain (Van Essen et al., 2012). In comparison, construction of a brain *functional* connectome has been in a far less advanced stage. Current efforts for a human functional connectome are focused on the resting-state only (e.g., the 1000 Functional Connectomes Project, http://fcon_1000.projects.nitrc.org). It is important to note that brain functional connectivity (FC) is dynamic and state-dependent. For example, performing a memory task, processing language information, being aroused emotionally, and listening to music can all elicit distinct patterns of FC, each of which can be considered a manifestation of the functional connectome. Furthermore, even for the same type of task, FC patterns may vary with a change of parameters. Therefore, whereas a completed structural connectome consists of definitive information of a finite number of projections among brain structures, the number of state-dependent FC pathways in a functional connectome greatly outnumbers that in the structural connectome. Although highly challenging, the time is ripe for the design and construction of functional connectomes based on the neuroinformatic tools developed for structural connectomes and the large volume of functional brain imaging data. Functional connectomes would allow comparison within and across experimental paradigms to refine current theories and to derive new theories about how the brain works at the circuit level.

Given the inherent complexity of brain functional connectomes, one approach is to compartmentalize the task and focus on a limited set of brain structures first. This approach has been taken in constructing a structural connectome for the retrosplenial cortex (Sugar et al., 2011) and the amygdala (Schmitt et al., 2012) in rodents. We propose here to choose the cortical midline structures (CMS) to start building a functional connectome for the rodent brain. We employ the term “functional connectome” to refer to a description of the functional relationships between subregions of the CMS, and the term “functional connectivity” to denote the symmetrical statistical association or dependency between individual brain regions (Bullmore and Sporns, 2009).

The CMS structures have attracted great research interest, both individually and as a whole. In rodents, from anterior to posterior, the CMS includes the prelimbic (PrL), cingulate area 1 (Cg1) and retrosplenial dysgranular (RSD) cortices dorsally, and medial orbital (MO), infralimbic (IL), cingulate area 2 (Cg2), and retrosplenial granular (RSG) cortices ventrally. Tract tracing studies have shown strong and reciprocal inter-regional anatomic projections and have suggested connectivity-based integration and segregation (Jones et al., 2005). Lesion and neurochemical mapping has also demonstrated *functional* integration and segregation (Vogt et al., 2013). The CMS structures are involved in a broad range of emotional, cognitive, attentional, and physiologic processes. Importantly, human neuroimaging findings in the past two decades show that the CMS is a central component of the default mode network, a network of structurally and functionally connected brain regions showing the highest metabolic level in the brain in the resting-state, but decreased metabolic rate when the brain is engaged in a task (Raichle et al., 2001). The CMS also contains candidate hubs such as the posterior cingulate cortex and medial prefrontal cortex with the highest level of FC of the resting-state network of the brain (Deco et al., 2011; Andrews-Hanna, 2012). This unique and central role of the CMS in the brain at rest further underscores the importance of better understanding of the functional organization within the CMS and between the CMS and other brain areas.

Human studies to date have just begun to systematically map FC at the subregion-level within the cingulate gyrus (Margulies et al., 2007; Habas, 2010; Yu et al., 2011). Brain FC has also been examined in rodents, including mice (Sif et al., 1989; Lee et al., 2009; Jonckers et al., 2011; White et al., 2011). Prior FC analysis of the CMS has typically selected a single seed region-of-interest (ROI) to represent an entire structure. Such an approach could mask subregion-level functional segregation as suggested by structural connectivity data. In the present study, we provide a more comprehensive examination of subregional FC within the anterior-posterior and dorsal-ventral axes of the CMS of the mouse. Furthermore, we sought to evaluate whether FC patterns of the CMS reported during fear in humans would parallel those observed in the mouse. CMS in the mouse have been proposed to model many of the cytoarchitectural and receptor binding characteristics of the human CMS (Vogt et al., 2013), however, little is known about its FC. Finally, because most imaging studies performed in mice have been performed in anesthetized animals, and because anesthesia can impact FC (Nallasamy and Tsao, 2011), we performed cerebral perfusion mapping in the current study in awake, nonrestrained mice. In our study, we applied perfusion mapping with autoradiographic methods, with FC calculated at a single time point using inter-subject, region-of-interest correlation analysis. This approach is similar to FC analyses performed in positron emission tomography (PET) data, but differs from the time-series correlation typically used in functional magnetic resonance imaging (fMRI). As such our analysis precludes evaluation of the dynamics of functional brain activation.

## Methods

### Animals

Male C57BL/6 mice were bred at the university vivarium from pairs obtained from Taconic (Taconic, Hudson, NY, USA). Mice had been backcrossed onto a C57BL/6 background for greater than 15 generations from an original mixed background [129/P1ReJ (ES cells), C57BL/6J and CD-1] (Bengel et al., 1998). Male mice were weaned at 3 weeks of age, housed in groups of 3–4 on a 12 h light/ 12 h dark cycle (lights on at 0600) until 3 months of age with direct contact bedding and free access to rodent chow (NIH #31M diet) and water. At the start of experimentation, animals were individually housed. All testing was conducted during the light phase of the light/dark cycle (0930–1430). All experimental protocols were approved by the Institutional Animal Care and Use Committee of the University of Southern California. Behavioral data and data of regional brain activation have been previously reported (Pang et al., 2011).

### Functional brain mapping

#### Surgery

Animals were anesthetized with isoflurane (2.0%). The ventral skin of the neck was aseptically prepared and the right external jugular vein was catheterized with a 1-French silastic catheter (SAI Infusion Technologies, Chicago, IL, USA), which was advanced 1 cm to the superior vena cava. The catheter was externalized through subcutaneous space to a dorsal percutaneous port. The catheter was filled with 0.01 mL Taurolidine-Citrate lock solution (SAI Infusion Technologies) and was closed with a stainless steel plug.

#### Conditioned fear- training phase

Fear conditioning experiments were conducted as previously described (Pang et al., 2011) at 3 days post-surgery. Animals were habituated to the experimental room for 30 min in their home cages. Thereafter, mice were placed in a Plexiglas box (22.5 × 21 × 18 cm) with a floor of stainless steel rods. The chamber was illuminated with indirect ambient fluorescent light from a ceiling panel (930 lx) and was subjected to background ambient sound (65 dB). After a 2-min baseline, the animals were presented a tone six times (30-s duration, 70 dB, 1000 Hz/8000 Hz continuous, alternating sequence of 250-ms pulses). Each tone was separated by a 1-min quiet period. In the conditioned fear group (body weight = 26 ± 0.5 g, age = 12.8 ± 0.3 wks, *n* = 13) each tone was immediately followed by a foot shock (0.5 mA, 1 s). Control animals (body weight = 26 ± 0.3 g, age = 12.4 ± 0.2 wks, *n* = 11) received identical exposure to the tone but without the foot shock. One minute following the final tone, mice were returned to their home cages.

#### Functional brain mapping during conditioned fear recall

Twenty-four hours after the training session, animals were placed in the experimental room for 1 h in their home cages. Thereafter, the animal's percutaneous cannula was connected to a tethered catheter containing the perfusion radiotracer ([^14^C]-iodoantipyrine, 325 μ Ci/kg bodyweight in 0.18 mL of 0.9% saline, American Radiolabelled Chemicals, St. Louis, MO, USA) and a syringe containing a euthanasia solution (50 mg/kg pentobarbital, 3M KCl). Animals were allowed to rest in a transit cage for 10 min prior to exposure to a novel behavioral cage (a cylindrical Plexiglas cage with a flat Plexiglas floor, dimly lit at 300 lx). Fear-conditioned and control animals received a 2-min exposure to the behavioral cage followed by a 1-min continuous exposure to the conditioned tone. One minute after the start of the tone exposure, the radiotracer was injected intravenously at 1 mL/min using a mechanical infusion pump (Harvard Apparatus, Holliston, MA, USA), followed immediately by injection of the euthanasia solution. This resulted in cardiac arrest within 5–10 s, a precipitous fall of arterial blood pressure, termination of brain perfusion, and death. Brains were rapidly removed and flash frozen in methylbutane over dry ice.

#### Autoradiography

Brains were sliced in a cryostat at −20°C into 20-μm coronal sections, with an inter-slice spacing of 140 μm. Slices were heat dried on glass slides and exposed to Kodak Ektascan diagnostic film (Eastman Kodak, Rochester, NY, USA) for 14 days at room temperature along with 12 [^14^C] standards (Amersham Biosciences, Piscataway, NJ, USA). Autoradiographs were then digitized on an 8-bit gray scale using a voltage stabilized light box (Northern Lights Illuminator, InterFocus Ltd., Linton, England) and a Retiga 4000R charge-coupled device monochrome camera (QImaging, Surrey, Canada). Cerebral blood flow (CBF) related tissue radioactivity was measured by the classic [^14^C]-iodoantipyrine method and used as a proxy measure of neuronal activation. In this method, there is a strict linear proportionality between tissue radioactivity and CBF when the data is captured within a brief interval (~10 s) after the tracer injection (Van Uitert and Levy, 1978; Jones et al., 1991).

#### Image preprocessing

Three-dimensional (3D) reconstruction has been described before (Nguyen et al., 2004). In short, regional CBF (rCBF) was analyzed on a whole-brain basis using statistical parametric mapping (SPM, version SPM5, Wellcome Center for Neuroimaging, University College London, London, UK). SPM, a software package developed for the analysis of human neuroimaging data (Friston et al., 1991), has recently been adapted by us and others for use in rodent brain autoradiographs (Nguyen et al., 2004; Lee et al., 2005; Dubois et al., 2008). A 3D reconstruction of each animal's brain was conducted using 69 serial coronal sections (starting at bregma +2.98 mm) with a voxel size of 40 × 140 × 40 μm. Adjacent sections were aligned both manually and using TurboReg, an automated pixel-based registration algorithm (Thevenaz et al., 1998). After 3D reconstruction, all brains were smoothed with a Gaussian kernel (FWHM = 120 × 420 × 120 μm). The smoothed brains from all groups were then spatially normalized to a smoothed reference brain (one “artifact free” brain). Following spatial normalization, normalized images were averaged to create a mean image, which was then smoothed to create the smoothed template. Each smoothed original 3D reconstructed brain was then spatially normalized into the standard space defined by the smoothed template (Nguyen et al., 2004). Voxels for each brain failing to reach a specified threshold in optical density (80% of the mean voxel value) were masked out to eliminate the background and ventricular spaces without masking gray or white matter. To account for any global differences in the absolute amount of radiotracer delivered to the brain, adjustments were made by the SPM software in each animal by scaling the voxel intensities so that the mean intensity for each brain was the same (proportional scaling).

### Pairwise inter-regional correlation analysis

**Figure 1:**
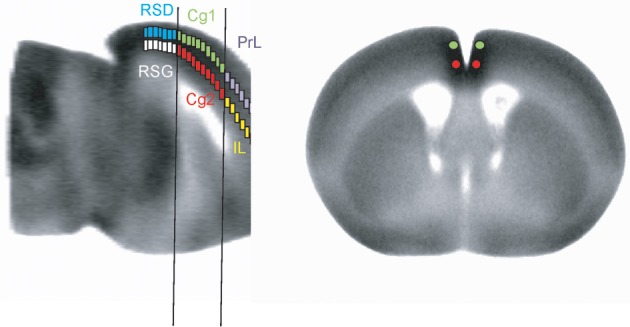
**Representation of region-of-interest selections (not to scale) in the cortical midline structures**.

Anatomical regions of interest (ROIs) in the CMS were sampled with a manually drawn circular ROI defined in MRIcro (version 1.40, http://cnl.web.arizona.edu/mricro.htm) on the template brain (Figure 1). ROI location was decided according to the anatomic parcellation defined in the Franklin and Paxinos mouse brain atlas (Franklin and Paxinos, 2007), and using the central sulcus, cortical surface, and corpus callosum as primary landmarks. Thirty-seven circular ROIs (100 μm in diameter) were selected bilaterally in 37 coronal slices (bregma +2.56 mm to −2.48 mm, 140-μm inter-slice distance, one ROI on each slice) across the dorsal structures of the CMS (PrL, Cg1, RSD). Additional 37 bilateral ROIs were selected in ventral structures (MO, IL, Cg2, RSG). Since two ROIs were selected at each bregma level, the most anterior part of Cg1 overlapping with PrL, and the most posterior part of IL overlapping Cg2 were not included in the analysis. Mean optical density of each ROI was extracted for each animal using the Marsbar toolbox for SPM (version 0.42, http://marsbar.sourceforge.net/). A pairwise inter-regional correlation matrix was calculated across animals for each group in Matlab (version 6.5.1, The MathWorks Inc., Natick, MA, USA). The matrices were visualized as heatmaps with Z-scores of Pearson's correlation coefficients color-coded. Statistical significance of between-group difference of a correlation coefficient was evaluated using the Fisher's Z-transform test (*P* < 0.05).

### Graph theoretical analysis

In graph theory, a network is defined as a set of nodes or vertices and the edges or lines between them (Bullmore and Sporns, 2009). Analysis was performed on networks defined by the above correlation matrices in the Pajek software (version 3.12, http://vlado.fmf.uni-lj.si/pub/networks/Pajek/) (De Nooy et al., 2011). Each ROI was represented by a vertex (node) in a graph, and two vertices with significant correlation (positive or negative) were linked with an edge. We used cluster analysis to delineate the organization of the CMS network. Hierarchical clustering based on dissimilarity was calculated in Pajek using the d1 dissimilarity index, which quantifies the difference in FC profile between each pair of ROIs. Results were visualized as dendrograms. In addition, a Kamada-Kawai algorithm was implemented to arrange the graph such that strongly connected regions were placed closer to each other, while weakly connected regions were placed further apart. The “energized” graphs further facilitated visualization and identification of the organizational characteristics of the CMS network.

### Seed correlation analysis

To evaluate functional segregation within the CMS, as well as to test the hypothesis that fear conditioning may have resulted in altered CMS functional connectivity profile, we applied seed-ROI correlation analysis. Unilateral seed ROIs—PrL (bregma +2.28 to +2.0 mm), IL (bregma +2.1 to +1.86 mm), Cg1 (bregma +0.88 to +0.6 mm), Cg2 (bregma +0.88 to +0.6 mm), RSD (bregma −0.94 to −1.22 mm), RSG (bregma −0.94 to −1.22 mm)—were hand drawn for the right hemisphere over the template brain as described above. Mean optical density of the seed ROIs was extracted for each animal. Correlation analysis was performed in SPM for each group using the seed values as a covariate. Threshold for significance was set at *P* < 0.05 at the voxel level and an extent threshold of 100 contiguous voxels. Regions showing significant correlations in rCBF with the ROI were considered functionally connected with the ROI.

## Results

### Pairwise inter-regional correlation analysis

**Figure 2:**
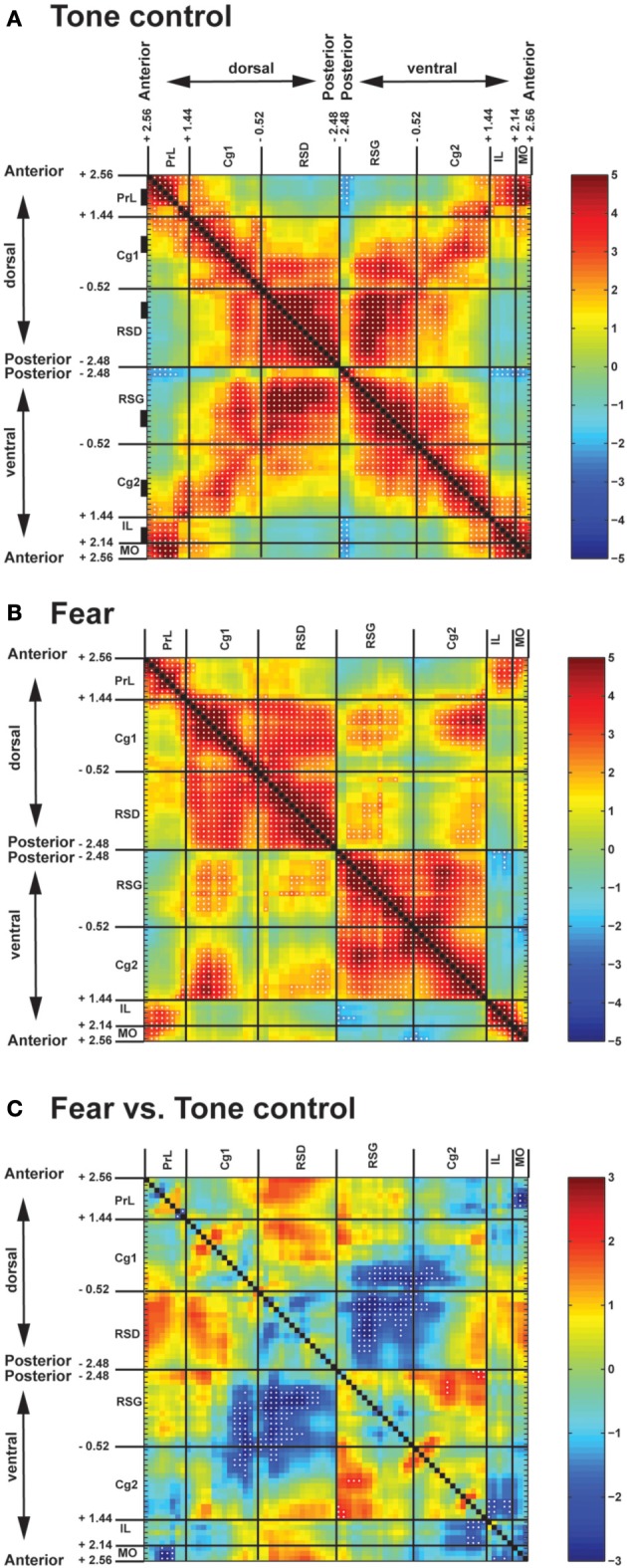
**Pairwise inter-regional correlation matrices showing functional connectivity among cortical midline structures of fear-conditioned and control mice. (A)** Control mice exposed to a neutral tone. **(B)** Fear-conditioned mice during auditory-cued fear recall. *Z*-scores of Pearson's correlation coefficients are color-coded. Each matrix is symmetric across the black diagonal line from upper-left to lower-right. Significant correlations (*P* < 0.05) are marked with white dots. **(C)** Statistical comparison of correlation coefficients between the fear-conditioned and the control group. The matrix of Fisher's *Z*-statistics represents differences in Pearson's correlation coefficients (*r*). Positive *Z*-values indicate greater *r* in the fear-conditioned group, while negative *Z*-values indicate smaller *r*. Significant between-group differences (*P* < 0.05) are marked with white dots. Numbers along the axes denote the anterior-posterior position in mm relative to the bregma. Black rectangles along the vertical axis in **(A)** denote anterior-posterior location of region-of-interests used in the seed correlation analysis. Abbreviations: Cg1, cingulate cortex area 1; Cg2, cingulate cortex area 2; IL, infralimbic cortex; MO, medial orbital cortex; PrL, prelimbic cortex; RSD, retrosplenial dystranular cortex; RSG, retrosplenial granular cortex.

Control mice when exposed to the neutral tone showed strong intra-regional FC in all CMS structures (Figure 2A, along the upper-left to lower-right diagonal). Along the anterior-posterior axis, short- to mid-range inter-regional FC connected neighboring structures. In particular, the anterior part of the Cg was functionally connected rostrally with PrL, MO, and IL, whereas the posterior part of the Cg was connected caudally with RSD and RSG. Long-range FC was missing, leaving the anterior (PrL, MO, IL) and posterior part (RSD, RSG) of CMS functionally disconnected. In contrast, along the dorsal-ventral axis, strong FC connected dorsal and ventral structures (PrL↔MO, PrL↔IL, Cg1↔Cg2, RSD↔RSG; Figure 2A, along the lower-left to upper-right diagonal).

Fear recall induced significant changes in the pattern of FC in the CMS (Figures 2B,C). Fear-conditioned mice compared to controls demonstrated significantly decreased FC along the dorsal-ventral axis (decreases in RSD↔RSG, posterior Cg1↔posterior Cg2, posterior Cg1↔RSG, RSD↔posterior Cg2). Increases in FC were noted primarily between Cg1 and RSD, and between Cg2 and RSG, resulting in a dorsal and a ventral cingulate-retrosplenial cluster with almost complete connections within each cluster. Results showed a similar pattern when examined separately in the left or the right hemisphere (data not shown).

### Graph theoretical analysis

**Figure 3:**
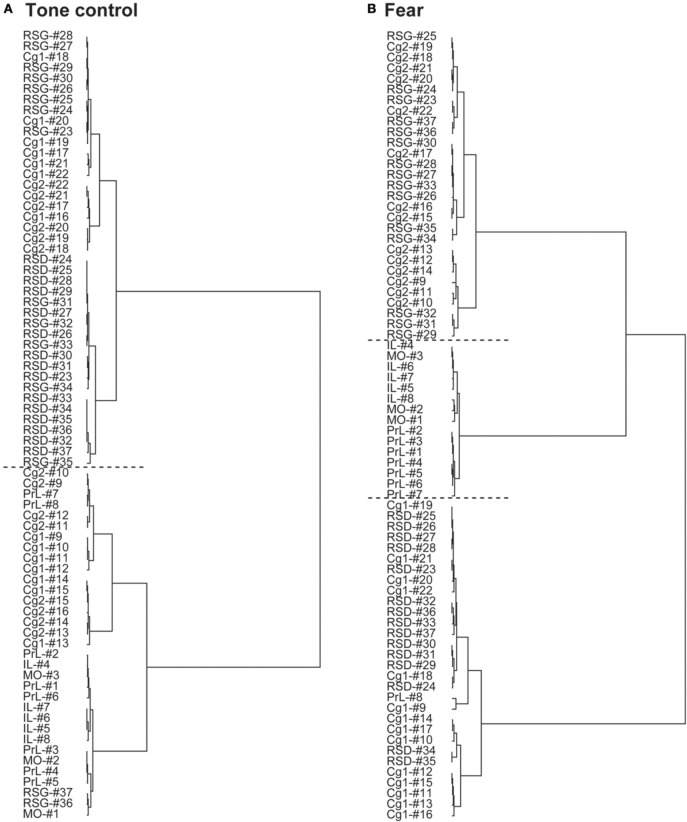
**Differences in hierarchical clustering between the control and fear-conditioned mice. (A)** Control mice exposed to a neutral tone. **(B)** Fear-conditioned mice during auditory-cued fear recall. The dendrograms show hierarchical clustering of the functional networks of the cortical midline structures. Dissimilarity between two regions-of interest (ROIs) is represented by the horizontal distance to their nearest joining point. Horizontal dashed lines are used to separate main clusters. Abbreviations: Cg1, cingulate cortex area 1; Cg2, cingulate cortex area 2; IL, infralimbic cortex; MO, medial orbital cortex; PrL, prelimbic cortex; RSD, retrosplenial dystranular cortex; RSG, retrosplenial granular cortex. The index numbers denote sequence of ROIs along the anterior-posterior axis such that #1 denotes the most anterior ROI at 2.56 mm anterior to the bregma and #37 the most posterior ROI at 2.48 mm posterior to the bregma. The inter-ROI distance along the anterior- posterior axis in the brain is 0.14 mm.

The dendrograms in Figure 3 show hierarchical clustering of the CMS functional network in control and fear-conditioned mice. Dissimilarity between two ROIs is represented by the horizontal distance to their nearest joining point. The control mice showed two main clusters: one anterior (PrL, MO, IL, anterior Cg1, and Cg2), the other posterior (RSD, RSG, posterior Cg1, and Cg2). The anterior cluster could be further divided into an anterior CMS group (PrL, MO, IL) and an anterior Cg group. The posterior cluster could also be divided into two groups, the first included posterior Cg and anterior RSG, while the second included RSD and posterior RSG. In contrast, fear-conditioned mice showed three main clusters: the anterior CMS (PrL, MO, IL), posterior dorsal CMS (Cg1, RSD), and posterior ventral CMS (Cg2, RSG).

**Figure 4:**
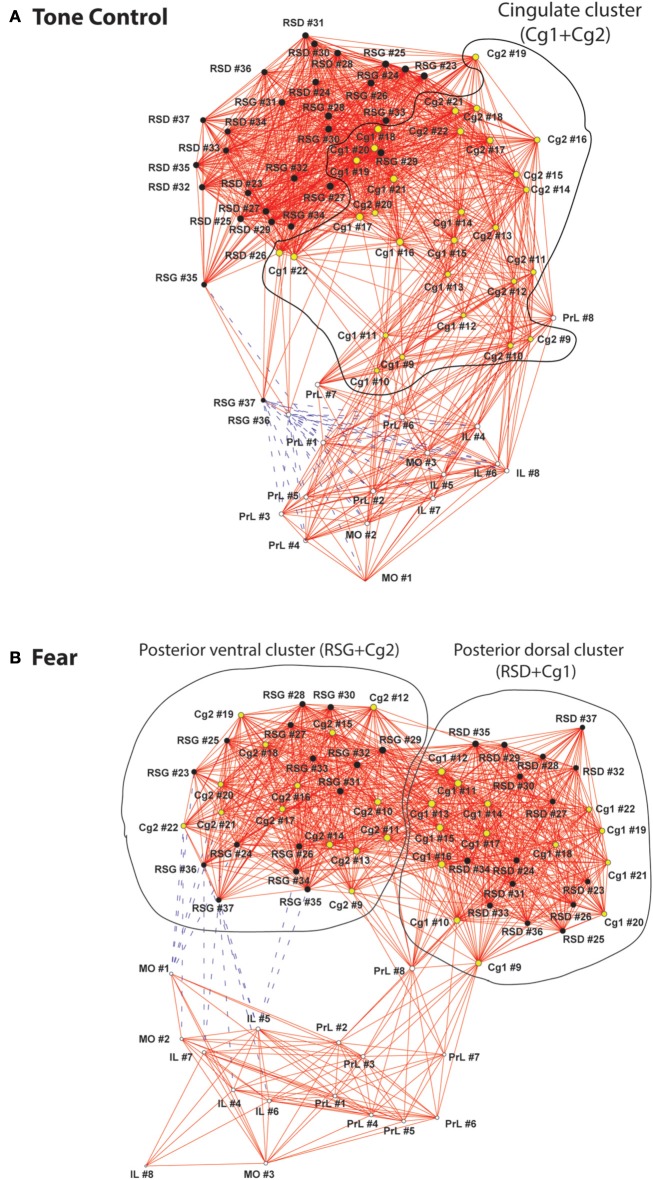
**Graph theoretical analysis of the functional networks of the cortical midline structures. (A)** In the control mice, relative location of regions of interest (ROIs) along the anterior-posterior axis was largely preserved topologically in the functional network. The cingulate cluster, which is circled and highlighted in yellow, connected anterior and posterior aspects of the cortical midline structures (CMS). **(B)** The fear-conditioned mice showed reorganization of the CMS functional network. Two distinct clusters are circled including a posterior dorsal and a posterior ventral cluster. The functional connectivity networks are represented with graphs, in which nodes (vertices) represent region of interests (ROIs) and edges represent significant correlations. Solid red lines denote significant positive correlations, whereas dashed blue lines significant negative correlations. The graphs were energized using the Kamada**–**Kawai algorithm that placed strongly correlated nodes closer to each other while keeping weakly correlated nodes further apart. The size of each node (in area) is proportional to its degree centrality, a measurement of the number of connections linking the node to other nodes in the network. Abbreviations: Cg1, cingulate cortex area 1; Cg2, cingulate cortex area 2; IL, infralimbic cortex; MO, medial orbital cortex; PrL, prelimbic cortex; RSD, retrosplenial dystranular cortex; RSG, retrosplenial granular cortex. The index numbers denote sequence of ROIs along the anterior-posterior axis, such that #1 denotes the most anterior ROI at 2.56 mm anterior to the bregma, and #37 the most posterior ROI at 2.48 mm posterior to the bregma. The inter-ROI distance along the anterior- posterior axis in the brain is 0.14 mm.

Organization of the CMS functional networks was further characterized with energized graphs (Figure 4). In the control mice, the relative location of ROIs along the anterior-posterior axis was largely preserved topologically in the functional network (Figure 4A). The cingulate cortices connected anterior CMS (PrL, MO, IL) and posterior CMS (RSD, RSG). The two most posterior RSG ROIs were connected directly to the anterior CMS through negative FC. Consistent to the cluster analysis results (Figure 3B), the fear-conditioned mice showed the same three clusters (Figure 4B). The posterior RSG was connected through negative FC to the ventral aspect of anterior CMS (IL, MO).

**Figure 5:**
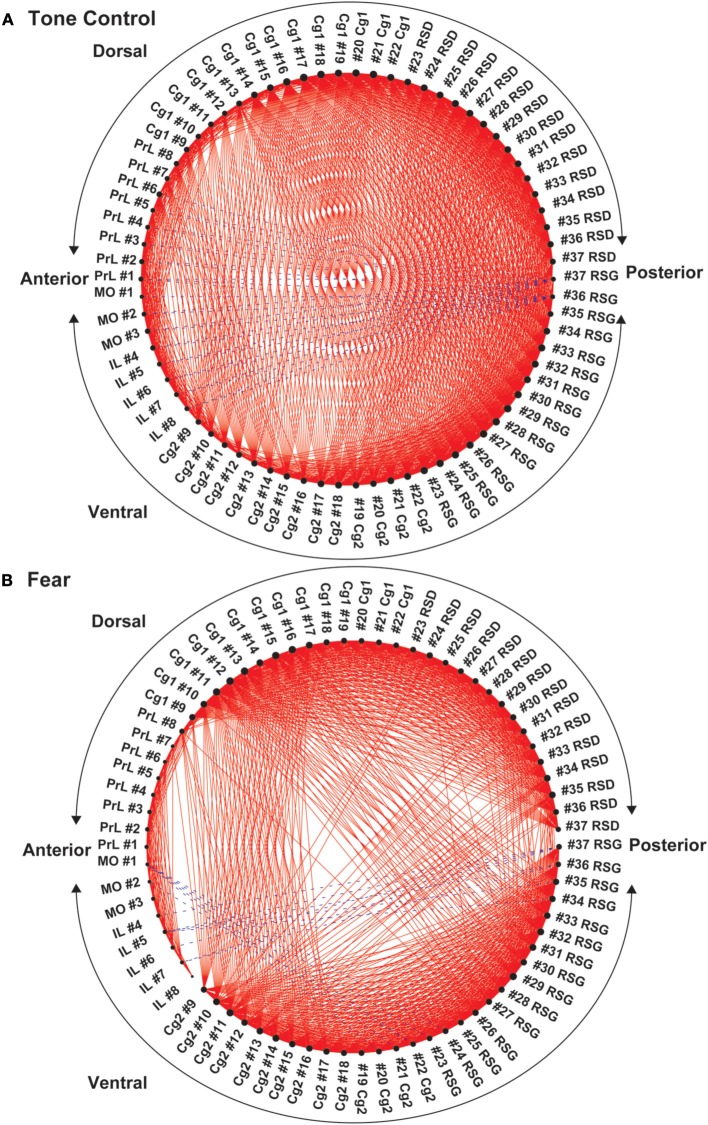
**Circular plot of graphs representing the functional networks of the cortical midline structures (CMS). (A)** In control mice, functional segregation was observed along the anterior-posterior axis between the anterior aspect (PrL, MO, IL, anterior Cg1, and Cg 2) and posterior aspect (RSD, RSG, posterior Cg1, and Cg2) of the CMS. **(B)** In the fear-conditioned mice, functional connectivity between the dorsal and ventral aspect of the CMs was greatly reduced. In each circular plot, regions of interest (ROIs) representing the dorsal CMS are arranged in the upper half of the circle, whereas ROIs representing the ventral CMS are arranged in the lower half. Abbreviations: Cg1, cingulate cortex area 1; Cg2, cingulate cortex area 2; IL, infralimbic cortex; MO, medial orbital cortex; PrL, prelimbic cortex; RSD, retrosplenial dystranular cortex; RSG, retrosplenial granular cortex. The index numbers denote sequence of ROIs along the anterior-posterior axis such that #1 denotes the most anterior ROI at 2.56 mm anterior to the bregma and #37 the most posterior ROI at 2.48 mm posterior to the bregma. The inter-ROI distance is 0.14 mm.

Functional segregation of the anterior CMS (PrL, MO, IL), particularly in the fear-conditioned mice, was further visualized in Figure 5. Also clearly shown was the functional disconnection between posterior dorsal (Cg1, RSD) and posterior ventral (Cg2, RSG) aspect of CMS in fear-conditioned mice.

### Seed analysis

**Figure 6:**
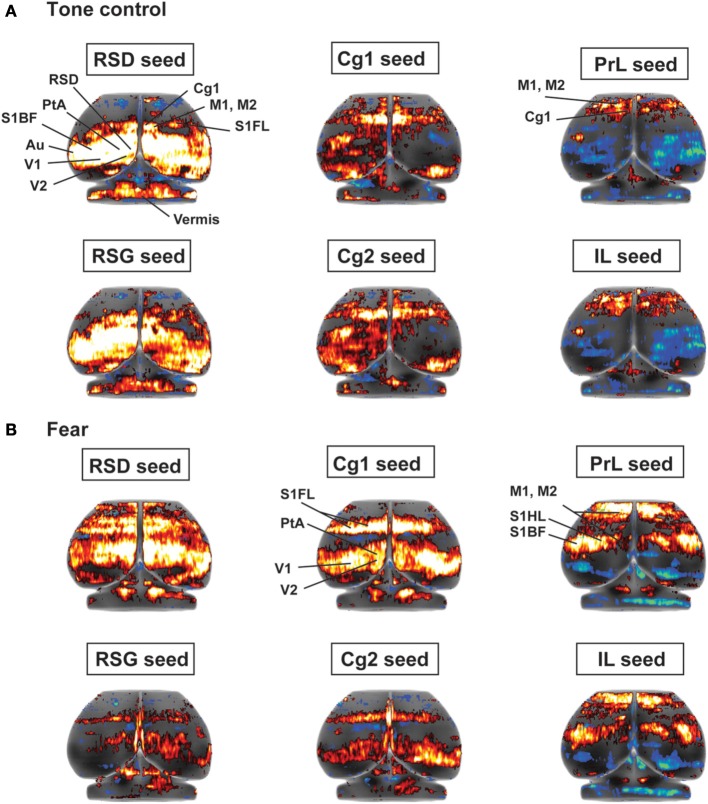
**Functional connectivity of the cortical midline structures (CMS) with the cerebral cortex. (A)** Control mice. **(B)** Fear-conditioned mice. Results of seed correlation analysis are plotted in a top-down view of the cerebral cortex. Voxel-wise correlation coefficients with the CMS seeds are color coded, with red and blue representing positive and negative correlation, respectively. Abbreviations for cortical structures: Au, auditory; Cg1, cingulate area 1; Cg2, cingulate area 2; IL, infralimbic; M1, primary motor; M2, secondary motor; MO, medial orbital; PrL, prelimbic; PtA, parietal association; RSD, retrosplenial dystranular; RSG, retrosplenial granular; S1BF, primary somatosensory, barrel field; S1FL, primary somatosensory, forelimb; S1HL, primary somatosensory, hindlimb; V1/V2, primary/secondary visual.

**Figure 7:**
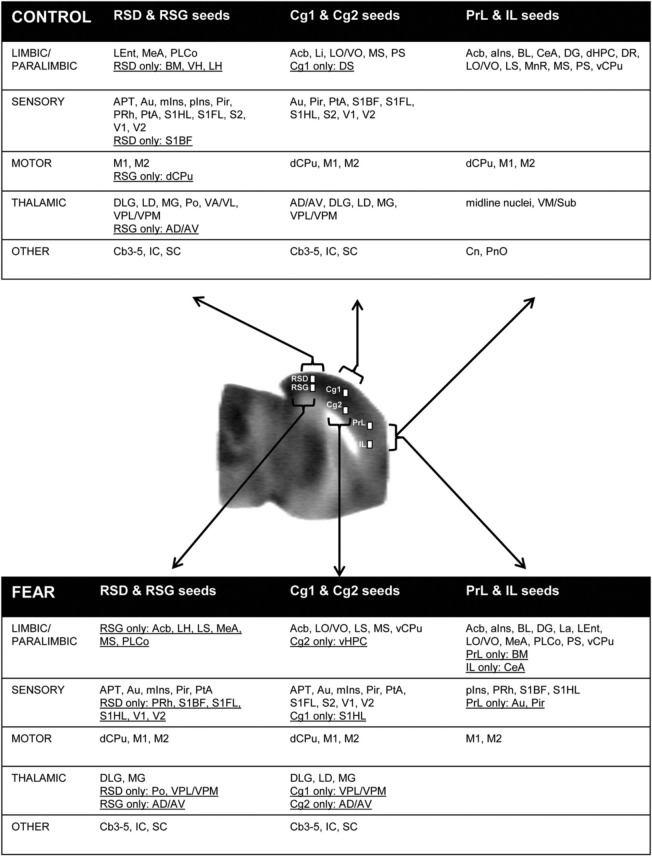
**Summary of positive functional connectivity for cortical midline structures with other brain areas.** Significant correlations are shown for seeds representing the right prelimbic, infralimbic, cingulate area 1, cingulate area 2, retrosplenial dysgranular and retrosplenial granular cortices. Brain areas that showed correlation to only one seed in each dorsal-ventral pair were underlined. Abbreviations: aIns, anterior insular cortex; APT, anterior pretectal area; Au, auditory cortex; AD/AV, anterior dorsal/ventral thalamic n.; BL, basolateral amygdalar n.; BM, basomedial amygdalar n.; Cb3–5, cerebellar lobules 3–5; CeA, central amygdalar n.; Cn, cuneiform n.; dCPu, dorsal caudate putamen; DG, dentate gyrus; DLG, lateral geniculate, dorsal; DR, dorsal raphe; DS, dorsal subiculum; IC, inferior colliculus; La, lateral amygdalar n.; LD, lateral dorsal thalamic n.; Lent, lateral entorhinal cortex; Li, linear raphe; LO/VO, lateral/ventral orbital cortex; LS, lateral septum); M1, primary motor cortex; M2, secondary motor cortex; MeA, medial amygdalar n.; mIns, mid insular cortex; MG, medial geniculate; MnR, median raphe; MS, medial septum; Acb, nucleus accumbens; vCPu, ventral caudate putamen; vHPC, ventral hippocampus; Pir, piriform cortex; PnO, pons; pIns, posterior insular cortex; PRh, perirhinal cortex; PS, parasubiculum; PtA, parietal association cortex; S1BF, primary somatosensory cortex, barrel field; S1FL, primary somatosensory cortex, forelimb; S2, secondary somatosensory cortex; SC, superior colliculus; V1/V2, primary/secondary visual cortex; VA/VL, ventral anterior/ventrolateral thalamic n.; VM/Sub, ventromedial/submedial thalamic n.; VPL/VPM, ventroposterolateral/ventroposteromedial thalamic n.

Seed FC analysis in control animals revealed a functional segregation such that anterior CMS (PrL and IL) showed a preferential positive connectivity to limbic/paralimbic structures, while posterior-most CMS (RSD, RSG) showed a preferential positive connectivity to sensory structures (Table 1, Figures 6A, 7). As a general trend, positive correlations for the anterior CMS would appear as negative or nonsignificant for the retrosplenial cortices. Likewise, positive correlations for the retrosplenial cortices would appear as negative or nonsignificant for the anterior CMS. Thus, for limbic/paralimbic structures, the PrL and IL showed positive correlations with the anterior insula (aIns), lateral and ventral orbital cortices (LO/VO), lateral and medial septa (LS, MS), amygdala (central n., CeA; basolateral n., BL), dorsal and median raphe (DR, MnR), nucleus accumbens (Acb), ventral caudate putamen (vCPu), dorsal hippocampus (dHPC), dentate gyrus (DG), and postsubiculum (PS). These correlations were either negative or nonsignificant for the retrosplenial seeds. Likewise, for sensory structures such as auditory cortex (Au), mid and posterior insula (mIns, pIns), primary somatosensory cortices (barrel field, S1BF; forelimb, S1FL; hindlimb, S1HL), secondary somatosensory cortex (S2), parietal association cortex (PtA), perirhinal and piriform cortices (PRh, Pir), visual cortices (V1, V2), the sensory thalamus (lateral genicular, dorsal, DLG; lateral dorsal, LD; medial geniculate, MG; ventral posterior lateral/ventral posterior media, VPL/VPM), anterior pretectal area (APT), inferior and superior colliculi (IC, SC), correlations were positive for RSD and RSG, but negative or nonsignificant for PrL and IL. While functional segregation was clearly noted along the anterior-posterior axis, no distinct segregation was noted along the dorsal-ventral axis. The cingulate (Cg1, Cg2) and retrosplenial cortices (RSD, RSG) showed overlapping FC patterns to the sensory areas, whereas the cingulate and the anterior CMS (PrL, IL) overlapped in their FC to the limbic/paralimbic areas. Of note, FC to primary and secondary motor cortices (M1, M2) showed significant positive correlations for all CMS seeds examined.

**Table 1:**
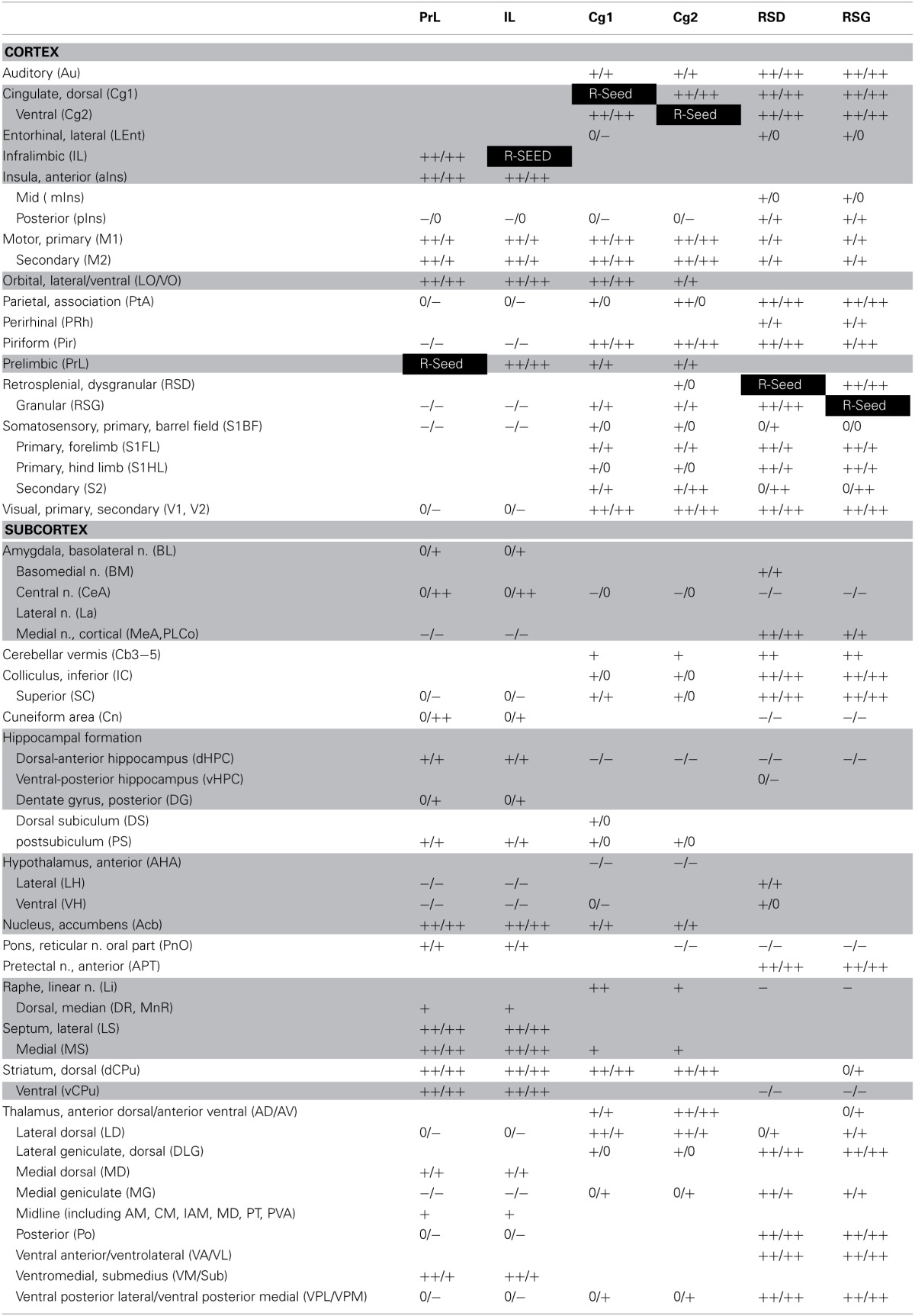
**Summary of seed correlation analysis results in the control mice**.

Functional connectivity of the cortical midline structures was analyzed using seed correlation for the right prelimbic (PrL), infralimbic (IL), cingulate area 1 (Cg1), cingulate area 2 (Cg2), retrosplenial dysgranular (RSD) and retrosplenial granular (RSG) cortices. Shown are significant left and right (L/R) positive (+) and negative (−) correlations with the seed (P < 0.05, clusters ≥ 100 voxels), with double signs denoting broadly represented correlations. “0” and blank cells denote the absence of significant correlations. Gray shaded cells highlight limbic/paralimbic areas. White text on a black background denotes the seed region.

While the anterior-posterior functional segregation was in general preserved in fear-conditioned mice, FC of the CMS to the limbic/paralimbic and sensory areas showed substantial changes (Table 2, Figures 6B, 7). In particular, fear conditioned recall broadened the FC of both PrL and IL to the amygdala, with newly emerged FC to the basomedial (BM) and lateral nuclei (La). Fear recall also changed the correlation of PrL with the medial amygdalar nucleus (PrL↔MeA) from negative to positive, while increasing the positive correlation of PrL↔CeA. The PrL and IL also showed new FC to the sensory and lateral entorhinal cortices (PrL, IL↔Au, pIns, PRh, S1BF, S1HL, LEnt, Figure 6B). Fear recall induced functional segregation along the dorsal-ventral axis, particularly in the retrosplenial cortices. Fear-conditioned mice compared to controls showed a loss of FC for RSG, but not RSD, to some of the sensory cortices (PRh, S1HL, S1HL, V1, V2, Figure 6B) and the sensory thalamus (Po, VPL/VPM). Whereas RSG became more broadly connected with the limbic/paralimbic areas (new FC with Acb, LH, LS, MS), RSD lost all its limbic/paralimbic FC.

**Table 2:**
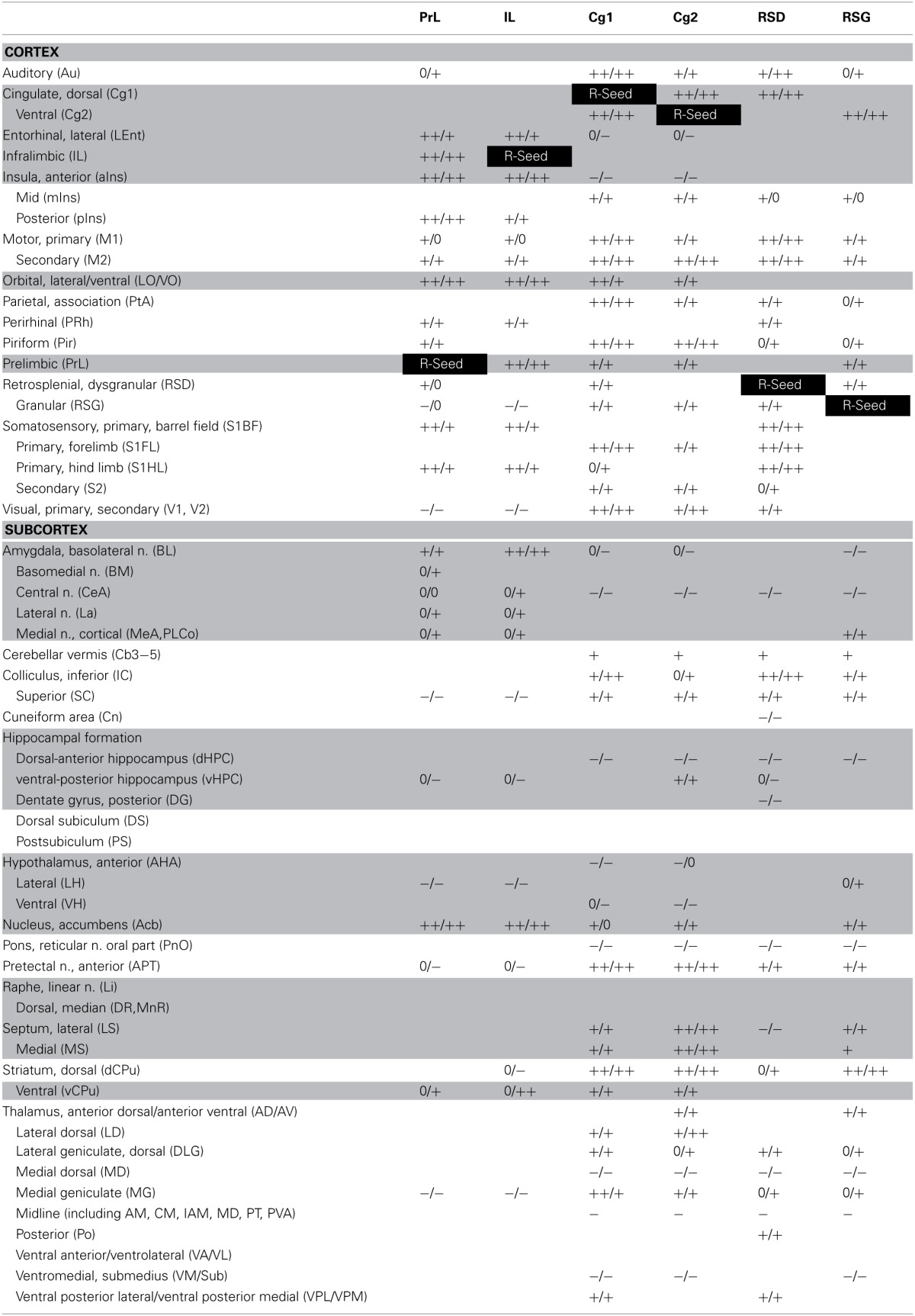
**Summary of seed correlation analysis results in the fear-conditioned mice**.

Functional connectivity of the cortical midline structures was analyzed using seed correlation for the right prelimbic (PrL), infralimbic (IL), cingulate area 1 (Cg1), cingulate area 2 (Cg2), retrosplenial dysgranular (RSD) and retrosplenial granular (RSG) cortices. Shown are significant left and right (L/R) positive (+) and negative (−) correlations with the seed (P < 0.05, clusters ≥ 100 voxels), with double signs denoting broadly represented correlations. “0” and blank cells denote the absence of significant correlations. Gray shaded cells highlight limbic/paralimbic areas. White text on a black background denotes the seed region.

## Discussion

To the best of our knowledge this is the first subregion-level functional connectivity analysis of the mouse cerebral midline structures. Our main findings include FC patterns among CMS structures and between CMS and the rest of the brain, as well as the impact of conditioned fear recall on these FC patterns.

### Functional connectivity among the cortical midline structures

In the control mice, pairwise correlation analysis showed strong intra-regional FC within each CMS structure (PrL, Cg1, RSD, IL, Cg2, and RSG) and strong inter-regional FC between contiguous structures along the anterior-posterior (PrL/MO/IL↔anterior Cg1/Cg2, posterior Cg1/Cg2↔RSD/RSG), as well as the dorsal-ventral axis (PrL↔MO/IL, Cg1↔Cg2, RSD↔RSG). In addition, the anterior (PrL, MO, IL) and posterior (RSD, RSG) aspect of CMS were functionally segregated. The fear-conditioned mice showed substantial functional reorganization. While the intra-regional FC was largely preserved, the anterior aspect of the CMS (PrL/MO/IL) became more segregated with the loss of most of its FC with the anterior Cg1/Cg2. Whereas FC was preserved for PrL↔MO/IL along the dorsal-ventral axis, FC was greatly reduced for Cg1↔Cg2, RSD↔RSG, posterior Cg1↔RSG and RSD↔posterior Cg2. In addition, FC was significantly enhanced along the anterior-posterior axis for Cg1↔RSD and Cg2↔RSG.

Graph theoretical analysis underscored these findings regarding functional integration and segregation of the CMS network. In the control mice, the CMS network showed remarkable topological organization along the anterior-posterior axis, with the mid CMS (Cg1/Cg2) connecting rostrally with the anterior CMS (PrL/MO/IL) and caudally with the posterior CMS (RSD/RSG). In contrast, the fear-conditioned mice showed increased functional integration dorsally between Cg1 and RSD, and ventrally between Cg2 and RSG, and increased functional segregation of the network into three clusters: the anterior (PrL/MO/IL), dorsal posterior (Cg1/RSD) and ventral posterior (Cg2/RSG) aspect of the CMS. It is important to note that in many cases, the purely data-driven graph theoretical analysis (Figures 3, 4) was able to segregate functional subnetworks in ways consistent with the underlying anatomic structure.

The structural connectivity across the CMS has been well documented (Jones et al., 2005; Vogt et al., 2013; Vogt and Paxinos, 2014). In the rat, Jones et al. (2005) reported reciprocal structural connections along the dorsal-ventral axis: between the PrL and IL, between the middle one third of the dorsal anterior cingulate (Cg1) and ventral anterior cingulate cortices (Cg2), and between dorsal (RSD) and ventral retrosplenial cortices (RSG). Along the anterior-posterior axis, the PrL provides axonal projections to the anterior part of Cg1 and Cg2, whereas the PrL/IL and anterior Cg1/Cg2 are largely disconnected from the other CMS structures. The posterior one third of Cg1 is reciprocally connected with the anterior aspect of both dorsal and ventral retrosplenial cortices (RSD/RSG) and receives projection from the posterior RSD/RSG. The posterior one third of Cg2 receives projection from the anterior RSD/RSG. Our FC results in the control mice concurred remarkably with these patterns of structural connectivity. Qualitatively similar patterns of structural connectivity among the CMS can be found in tract tracing data published online by the Mouse Connectome Project (http://www.mouseconnectome.org/), and the mouse connectivity database in the Allen Brain Atlas (http://connectivity.brain-map.org/).

### Functional connectivity of the cortical midline structures with other brain areas

Seed FC analysis in control animals revealed a functional segregation such that anterior-most structures (PrL and IL) showed a preferential positive connectivity to limbic/paralimbic areas, while posterior structures (RSD, RSG) showed a preferential positive connectivity to sensory areas. Furthermore, the sign of correlation (positive or negative) was often reversed for the anterior compared to the posterior CMS structures with regard to the limbic/paralimbic and sensory areas. Thus, the PrL and IL showed positive correlations with limbic/paralimbic areas, including the anterior insula, septum (lateral, medial), central and basolateral nuclei of the amygdala, nucleus accumbens, and dorsal hippocampus, whereas these limbic/paralimbic areas showed negative or nonsignificant correlations with the RSD and RSG. Likewise, for sensory areas such as the somatosensory cortices (S1BF, S1HL, S1FL, S2), parietal association cortex, visual cortices, auditory cortex, mid and posterior insula, the sensory thalamus (ventroposterior lateral/medial, medial geniculate), anterior pretectal nucleus, and colliculi (inferior, superior), correlations were positive for the RSD and RSG seeds, but negative or nonsignificant for the PrL and IL seeds. The above findings are consistent with the divergent roles of anterior medial prefrontal cortex and that of the retrosplenial cortex, with the former playing a role in the regulation of limbic activity (Margulies et al., 2007; Horn et al., 2010; Ichesco et al., 2012; Connolly et al., 2013; Klavir et al., 2013), and the latter receiving and integrating early-processed sensory information (Vann et al., 2009).

This functional segregation was in general preserved in fear-conditioned mice. In addition, fear conditioned recall broadened the FC of the PrL and IL to the amygdala, with new FC to the basomedial and medial nuclei. These results are consistent with the existent structural connectivity of the PrL and IL with the amygdala, as well as brain mapping studies suggesting their functional coactivation in the conditioned-fear paradigm (Cassell et al., 1989; Singewald et al., 2003; Holschneider et al., 2006; Knapska and Maren, 2009; Lehner et al., 2009; Sotres-Bayon et al., 2012). The fear-conditioned mice also showed new positive correlations between PrL/IL and sensory areas, including somatosensory cortices (S1BF, S1HL, PRh, pIns), auditory cortex (PrL only) and piriform cortex (PrL only), suggesting integration and processing of sensory information by the PrL/IL during fear recall. Altered FC of the retrosplenial cortices was observed in the fear-conditioned mice. The RSG was predominantly connected with the limbic/paralimbic areas, whereas the RSD was predominantly connected with the sensory areas. The significance of this shift in FC pattern remains to be further investigated.

The afferent and efferent projections of the CMS structures have been a subject of extensive research (Domesick, 1969; Sesack et al., 1989; Vertes, 2004; Hoover and Vertes, 2007; Sugar et al., 2011). While it is beyond the scope of this report to compare functional and structural connectivity of the CMS in detail, it is important to note that a functional connectome reflects the dynamic, state-dependent recruitment of the underlying structural network.

### Translational aspects

One widely accepted theoretical construct of the subdivisions of the human cingulate gyrus is the four-region model (Vogt et al., 2013), consisting of the anterior cingulate cortex (ACC; s, subgenual; p, pregenual), the midcingulate cortex (MCC; a, anterior; p, posterior), the posterior cingulate cortex (PCC; d, dorsal; v, ventral), and the retrosplenial cortex (RSC). This midline cortex shows evolutionary expansion across species, with increasing complexity as one progresses from rodents to nonhuman primates to humans (Vogt et al., 2013). PrL and IL in rodents appear to be homologous to primate pregenual ACC and subgenual ACC, respectively (Vogt et al., 2013; Vogt and Paxinos, 2014), although PrL may also show some features of primate dorsolateral prefrontal cortex (for further discussion see Uylings et al., 2003), and IL features of primate orbitomedial cortex (for further discussion see Vertes, 2006). There are no posterior cingulate areas in rodents, and posterior CMS is composed entirely of retrosplenial cortex, which is proportionally much larger in rodents than in humans (Vann et al., 2009; Vogt et al., 2013; Vogt and Paxinos, 2014). Hence, in rodents, the CMS is best described by a three-region model (Vogt and Paxinos, 2014), with key similarities of structural connectivity for intra-cingulate connections for humans, primates and rodents (Vogt et al., 2013).

Functional specialization of the cingulate gyrus has been explored in human subjects by Margulies et al. (2007) who examined resting-state FC patterns for 16 ACC seed regions. Their results demonstrated strong anterior-posterior and dorsal-ventral functional specialization of the ACC, and highlighted the negative relationships between rostral ACC-based affective networks and caudal ACC-based frontoparietal attention networks (Margulies et al., 2007). Habas (2010) mapped the FC patterns of the human rostral and caudal cingulate motor areas (located just under the pre-supplementary and supplementary motor areas), and found that activity in the rostral cingulate motor area was more correlated with activity in prefrontal, orbitofrontal, and language-associated cortices, whereas the caudal cingulate motor area correlated more closely with sensory cortex (Habas, 2010). More recently, Yu et al. (2011) examined functional connectivity of the human cingulate cortex using the four-compartment model (Yu et al., 2011). They found that the subgenual ACC and pregenual ACC were involved in an affective network, while being negatively correlated with a sensorimotor network. In the MCC, however, the anterior MCC was correlated with the sensorimotor network and negatively correlated with the affective network, whereas the posterior MCC only correlated with the sensorimotor network. The dorsal PCC and ventral PCC were involved in the default-mode network and were negatively correlated with the sensorimotor network. In contrast, the RSC was mainly correlated with the PCC and thalamus.

Our findings in the control mice parallel these human findings in general. The anterior CMS (PrL and IL) in the mouse showed a preferential FC to limbic/paralimbic areas, while mid (Cg1, Cg2) and posterior CMS (RSD, RSG) showed greater connectivity to sensory areas. Furthermore, the PrL and IL showed negative correlations with some sensory areas, whereas the cingulate and retrosplenial cortices showed negative correlations with some limbic/paralimbic areas. Differences were noted in the FC of the retrosplenial cortices, with the mouse showing broader FC with sensorimotor regions than that reported in the PCC in humans (Yu et al., 2011). This may reflect the fact that the rodent retrosplenial cortex is proportionally larger than in humans, and contains areas of cortex not represented in the human PCC (Vann et al., 2009). Of note, the retrosplenial cortex showed broad FC with thalamic nuclei in mice, which correlates with strong FC between these regions observed in humans (Yu et al., 2011). Finally, in agreement with prior work in human subjects, fear broadened FC of anterior CMS to the amygdala and to somatosensory areas, suggesting integration and processing of both limbic and sensory information (Hariri et al., 2003; Stein et al., 2007; Cullen et al., 2011; Robinson et al., 2012; Motomura et al., 2013; Prater et al., 2013).

### Methodological considerations

We applied pairwise inter-regional correlation analysis to autoradiographic CBF data to investigate brain functional connectivity (Wang et al., 2011, 2012). This is a well-established method, which has been applied to analyze rodent brain mapping data of other modalities, including autoradiographic deoxyglucose uptake (Soncrant et al., 1986; Barrett et al., 2003), cytochrome oxydase histochemistry (Fidalgo et al., 2011; Padilla et al., 2011), activity regulated genes (c-fos) (Wheeler et al., 2013), and fMRI (Schwarz et al., 2007). In these studies, correlations are calculated in an inter-subject manner, i.e., across subjects within a group. Hence, perfusion mapping using autoradiographic methods presents a “snap-shot” of brain activity at a single point in time, which in the case of the current study corresponded to a several second time window occurring 1 minute following exposure to the tone. Thus, our methods preclude analysis of the dynamics of functional brain activation. This approach is different from the intra-subject cross correlation analysis often used on fMRI time series data (Pawela et al., 2008; Magnuson et al., 2010; Liang et al., 2011) or that typically performed in electrophysiologic recordings (Scholvinck et al., 2013). Caution needs to be taken comparing FC results between different brain imaging modalities and between different analytic methods (Di et al., 2012; Buckner et al., 2013; Hutchison et al., 2013; Scholvinck et al., 2013; Wehrl et al., 2013).

What has become increasingly clear is that FC may occur at different time scales ranging, for example, from milliseconds in electrophysiologic studies, to seconds in fMRI and minutes in PET (Di et al., 2012; Scholvinck et al., 2013; Wehrl et al., 2013). Although the existence of a flow/metabolism coupling to neural activity is well accepted, and indeed forms the basis of the majority of functional brain mapping studies, it is true that the exact relationship between neuronal activity, regional CBF and metabolism, as well as the role of vascular distribution and architecture remains a question of debate (Gsell et al., 2000; Keri and Gulyas, 2003; Van Zijl et al., 2012), and the relationship between dynamic neurometabolic coupling and more static measures of regional covariance remains largely unresolved. Different analytic tools have been adapted to allow the determination of functional associations, either by accounting for the temporal aspects of time series or, as in the current study, by modeling the system over the entire experimental period independent of the temporal order (Stephan, 2004). Honey et al. (2007) who explored the network structure of cerebral cortex on multiple time scales reported that at the slowest time scale (minutes), the aggregate strength of functional couplings between regions is, on average, a good indicator of the presence of an underlying structural link (Honey et al., 2007). At faster time scales significant fluctuations are observed in the strength of functional coupling. Recent work has compared FC calculated using inter-subject, region-of-interest correlation analysis of ^18^fluorodeoxyglucose PET data and that using time-series correlation analysis of fMRI data (Di et al., 2012; Wehrl et al., 2013). These methods differ in their temporal scales, ranging from minutes for the PET images to seconds for the fMRI images. Findings suggest that in general the two methods generate comparable results with regards to core regions. However, differences in the time scales of data sampling may result in the differential recruitment of ancillary regions, and this effect may be accentuated in studies in which subjects receive an ongoing active stimulation. Future efforts at delineating a functional connectome will need to evaluate FC at multiple time scales to better address the issue of state vs. trait related changes.

It is important to remember that while correlation based analyses provide information about functional connectivity, they do not directly address causal relationships. Thus, it is possible that functional connectivity may arise even in the absence of a direct structural connection through functional linkages across a shared secondary node. However, while indirect interactions can account for some functional linkages, current evidence suggests that topological parameters are generally conserved between structural and functional networks (Bullmore and Sporns, 2009). Our approach to studying FC in the mouse brain appears reasonable and consistent with the current theoretical understanding of functional connectivity as long as one understands that it does not address causality or directionality of individual connections, and that it is conceivable that covariance between two nodes in a circuit may occur in the absence of their direct structural connectivity.

The results of our pairwise correlation analysis highlight the general challenge inherent in the interpretation of any ROI analysis—that is how representative is the selected ROI for assessing the functional connectivity of the structure of interest as a whole? An ROI defined either too large or too small relative to the actual extent of regional activation may result in loss of statistical power. For brain structures with complex spatial patterns of afferent and efferent projections, defining appropriate ROIs may be particularly difficult. A strength of our study was its unbiased approach of ROI selection across sequential coronal slices of the 3D midline cortex of the mouse. This unbiased approach allowed us to detect functional segregation of these regions without the limitations of pre-specified ROIs. In our study, the cingulate cortex (Cg1, Cg2) was itself functionally segregated such that the anterior half correlated more strongly with PrL, MO and IL, whereas the posterior half correlated more strongly with RSD and RSG. However, while our seed analysis (using pre-specified ROIs) suggested that the cingulate cortex had a pattern of functional connectivity that was intermediate between that of the anteriormost and posteriormost CMS, it is likely that an individual seed placed at different locations within the cingulate would result in progressively different FC patterns.

## Conclusion

Our study provided information on the functional connectivity pattern of the CMS at a mesoscopic level. Thus, while FC as implemented in the current study was not specified at a level that allowed one to distinguish between different processes at synaptic, cellular, columnar or laminar levels, it did allow one to model context-dependent changes at the level of large neural populations. Functional integration and segregation noted in our study paralleled reports of structural connectivity of CMS in the rodent, and were in general consistent with reports of functional connectivity in humans using fMRI. The subregion-level approach to defining individual functional units and constructing macro- to mesoscopic level connectomes for neural systems such as the CMS offered a balanced solution that facilitated comparison with structural connectivity data. Differences in FC between the control and fear-conditioned mice highlighted the state-dependence of brain functional connectome, and the importance of evaluating and comparing the functional connectome across states. Organizational principles learned from animal models at the macro- and mesoscopic level (brain regions/subregions and pathways) will not only inform future work at the microscopic level (single neurons and synapses) but may have translational value to advance our understanding of human brain structure and function, as well as of animal models of human cerebral pathology (Lynch et al., 2013).

### Conflict of interest statement

The authors declare that the research was conducted in the absence of any commercial or financial relationships that could be construed as a potential conflict of interest.
